# Novel group of tyrosyl-DNA-phosphodiesterase 1 inhibitors based on disaccharide nucleosides as drug prototypes for anti-cancer therapy

**DOI:** 10.1080/14756366.2018.1509210

**Published:** 2018-09-07

**Authors:** Anastasia O. Komarova, Mikhail S. Drenichev, Nadezhda S. Dyrkheeva, Irina V. Kulikova, Vladimir E. Oslovsky, Olga D. Zakharova, Alexandra L. Zakharenko, Sergey N. Mikhailov, Olga I. Lavrik

**Affiliations:** a Institute of Chemical Biology and Fundamental Medicine, Siberian Branch of the Russian Academy of Sciences, Novosibirsk, Russian Federation;; b Department of Natural Sciences, Novosibirsk State University, Novosibirsk, Russian Federation;; c Engelhardt Institute of Molecular Biology, Russian Academy of Sciences, Moscow, Russian Federation

**Keywords:** Disaccharide nucleosides, tyrosyl-DNA phosphodiesterase 1, TDP1 inhibitor, topotecan

## Abstract

A new class of tyrosyl-DNA phosphodiesterase 1 (TDP1) inhibitors based on disaccharide nucleosides was identified. TDP1 plays an essential role in the resistance of cancer cells to currently used antitumour drugs based on Top1 inhibitors such as topotecan and irinotecan. The most effective inhibitors investigated in this study have IC_50_ values (half-maximal inhibitory concentration) in 0.4–18.5 µM range and demonstrate relatively low own cytotoxicity along with significant synergistic effect in combination with anti-cancer drug topotecan. Moreover, kinetic parameters of the enzymatic reaction and fluorescence anisotropy were measured using different types of DNA-biosensors to give a sufficient insight into the mechanism of inhibitor’s action.

## 1. Introduction

Oncological diseases are one of the most frequent causes of human mortality in the world. According to the WHO’s International Agency for Research on Cancer (IARC), about 14 million cases of cancer are registered each year and more than half of them are fatal^1^. In the recent decades, the method of targeted cancer therapy is gaining popularity as one of the most effective mechanisms for suppressing growth of cancer cells^2^^,^^3^. A promising therapeutic target for the treatment of oncological diseases is the DNA repair enzyme tyrosyl-DNA-phosphodiesterase 1 (TDP1)^4–6^.

TDP1 catalyses the removal of both naturally and chemically induced 3′-DNA adducts^5^. In addition, TDP1 is involved in the repair of an oxidative damage in mitochondrial DNA^7^. One of the natural substrates for TDP1 is the covalent complex of another enzyme – topoisomerase 1 (Top1) with 3′-end of a DNA strand^8^. During chemotherapy of recurrent cancer and persistent tumours (colon, cervix, ovaries, lungs) this complex is stalled and stabilised by clinically used Top1 inhibitors such as natural antitumour compound camptothecin analogues: topotecan and irinotecan^9^^,^^10^. Accumulation of the resulting irreversible Top1–DNA cleavage complexes can lead to the formation of double-strand breaks following a collision of the replication and transcription machinery and, as a consequence, cell death^11^. Normally, such complexes are repaired by either TDP1^8^^,^^12^ or endonuclease pathway^13^^,^^14^, resulting in the reducing of the effectiveness of anti-cancer drugs. It is important to note that TDP1 post-translational regulation by ataxia-telangiectasia-mutated (ATM) and DNA-dependent protein kinase (DNA-PK) play a significant role in DNA repair in response to camptothecin-induced double-strand breaks and cell survival^15^. The hypothesis that TDP1 is responsible for the drug resistance of some cancers is confirmed by several reports about TDP1 knockout mice and human cell lines with mutation in the TDP1 gene that have shown hypersensitivity to camptothecin^16^^,^^17^. Conversely, in cells with an elevated level of TDP1 expression, camptothecin causes less DNA damage^18^^,^^19^. In this regard, inhibition of TDP1 can enhance the effectiveness of such Top1 inhibitors^9^^,^^20^. The combination of drugs acting on Top1 and TDP1 can significantly increase the effectiveness of chemotherapy and allow to reduce total toxicity on the organism through reducing the therapeutic dose of these anti-cancer drugs.

In terms of drug design, analogues of natural compounds are promising substances as enzyme inhibitors since they often have biological activity and a wide chemical diversity, which allows performing directional modification of the compounds in order to enhance their inhibitory activity^21^. In this context, disaccharide nucleosides and their derivatives are quite attractive substances for research and further development of drugs. Disaccharide nucleosides belong to an important group of natural compounds which demonstrates a broad spectrum of biological (including antibacterial, fungicidal, herbicidal, antitumour, and antiviral) activities^22–25^. To date, about a hundred disaccharide nucleosides and related derivatives have been isolated from different natural sources. These compounds contain an extra carbohydrate residue linked to one of the nucleoside hydroxyl groups *via* an *O*-glycosidic bond that makes their properties similar to those of carbohydrates and nucleosides and can be synthesised by one of two routes: (1) by coupling of a protected disaccharide with a heterocyclic base derivative or (2) by the formation of an *O*-glycosidic bond between a nucleoside carrying one free hydroxyl group and an activated monosaccharide^22^.

Moreover, the advantage of disaccharide nucleosides is that they are apparently capable to easily penetrate through both plasma and nuclear membranes of cells using a system of nucleoside transporters that is similar to the transport system of different antiviral (acyclovir, zidovudine, etc.) and antitumour drugs (cytarabine, cladribine, gemcitabine) based on nucleosides^26^. Also, it is known that this class of substances demonstrates sufficiently low own cytotoxicity^27–28^ that is important for the development of drugs for combination therapy.

Recently it was shown, that some pyrimidine disaccharide derivatives inhibit poly(ADP-ribose)polymerase-1 (PARP-1), a key enzyme of DNA repair^28^^,^^29^. Moreover, in the studies^30^^,^^31^ series of nicotinamide adenine dinucleotide (NAD^+^) mimetics, which comprise morpholino analogues of nucleosides were synthetised to inhibit PARP-1 enzyme. Here we describe a series of disaccharide nucleosides as a novel class of tyrosyl-DNA phosphodiesterase 1 (TDP1) inhibitors. To determine the inhibitory effect of the tested compounds and evaluate their biological properties for the isolated recombinant human TDP1 enzyme, we performed extensive fluorescence screening, steady-state kinetic experiments, and anisotropy measurement. We also determined the cytotoxicity of the most effective inhibitors and their synergistic effect in combination with anti-cancer drug topotecan.

## 2. Materials and methods

### 2.1. Synthesis of disaccharide nucleosides

Compounds **1–49** (Table 1) were prepared according to the literature procedures: **1, 3–4**^32–37^, **2**^38^, **5–11**^32^, **12**–**21**^39^, **22–23**, **34**^40^, **24–27**^41^^,^^42^, **32–33**^41–44^, **35–37**^41^^,^^42^, **28–29, 38–42**^28–29^, **30–31**^40^, **43–49**^43^^,^^44^. The presence of fluorine atoms in position 5 of pyrimidine residue in compounds **19**, **20**, **49** is confirmed by spin-spin coupling constants between ^19 ^F and ^1^H in ^1^H-NMR spectra (*J*_H-F_) and between ^19 ^F and ^13 ^C in ^13^C-NMR spectra (*J*_C-F_). ^1^H-NMR spectra of the fluorinated nucleosides **19**, **20**, **49** are complicated by the presence of two ^19^F-^1^H couplings: ^3^*J*_H6-F_–6.5 Hz, ^5^*J*_H1′-F_–1.5 Hz. In the ^13^C-NMR spectra, three types of coupling constants *J*_C-F_ are present, which are characteristic of fluorinated uracil derivatives: ^1^*J*_C-F_–230 Hz (5-C-atom), ^2^*J*_C-F_–26 Hz (4-C  =  O group), and ^2^*J*_C-F_–34 Hz (6-C-atom). An important characteristic for 5-iodouracil derivatives **39**–**42**, **48** is a strong displacement of a C-5 signal in ^13^C-NMR towards strong magnetic field (72.69 ppm) in a comparison with C-5 in uracil and thymine analogues (110–100 ppm). Partition coefficients (logP) were calculated using a program Instant J. Chem (ChemAxon).

**Table 1.** Structural formulas and inhibitory activities for the investigated disaccharide nucleosides.

*R_x_: substituting group, in case of “-“ R = H; Ade^Bz^: N^6^-Benzoyladenine-9-yl; Gua^i-But^: N^2^-Isobutyrylguanine-9-yl; Cyt^Bz^: N^4^-Benzoylcytosine-1-yl; 5-F-Ura: 5-Fluorouracil-1-yl; 5-I-Ura: 5-Iodouracil-1-yl.

### 2.2. Preparation of human recombinant TDP1 and TDP1 mutant form

The recombinant tyrosyl-DNA phosphodiesterase 1 and the mutant form of TDP1 with substitution (H493R) were expressed in the E. coli system (plasmids pET16B-TDP1 and pET16B-SCAN1 were provided by Dr. K.W. Caldecott, University of Sussex, United Kingdom and by Dr. S. El-Khamisy, University of Sheffield, United Kingdom) and purified according to the previously described technique^45^.

### 2.3. Fluorophore-quencher containing oligonucleotides

**Figure 1.**
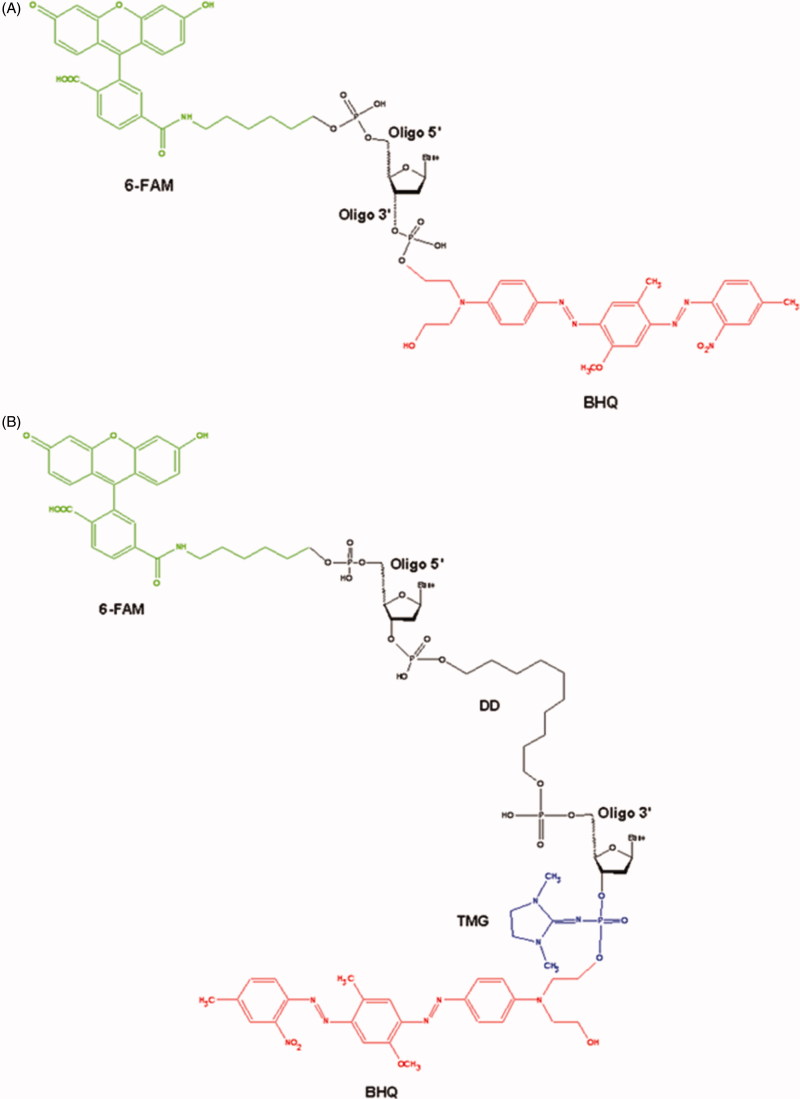
The main structural blocks of the single-stranded (A) and the hairpin (B) oligonucleotides.

Two types of DNA oligonucleotides containing fluorophore and fluorescence quencher were developed in the Laboratory of bioorganic chemistry of enzymes and synthesised in the Laboratory of biomedical chemistry at the Institute of Chemical Biology and Fundamental Medicine, Novosibirsk, Russia:

(1) The single-stranded oligonucleotide (5′-[FAM] AAC GTC AGG GTC TTC C [BHQ]-3′) containing fluorophore at the 5′-end (6-FAM) and a Black Hole Quencher 1 (BHQ) at the 3′-end (Figure 1(A)).

(2) The hairpin oligonucleotide (5′-[FAM] GGA AGA [DD] TCT TCC-p*- [BHQ]-3′). This oligonucleotide contains 6-FAM at the 5′-end, BHQ at the 3′-end, decanediol (DD) residue at the inflection point and the tetramethylphosphorylguanidine (TMG) residue attached to the phosphate group at the 3′-end (p*) (Figure 1(B))^46^.

### 2.4. Real-time detection of TDP1 activity

Conditions and details for our real-time detection of TDP1 activity using the single-stranded and hairpin fluorescent biosensors were reported in our previous work^47^. Briefly, the approach consists in fluorescence intensity measurement of the reaction of quencher removal from the fluorophore-quencher coupled DNA-oligonucleotide catalysed by TDP1 in the presence of inhibitor and in the absence of it (the control samples contained 1% DMSO). Reaction mixtures (200 µl) contained TDP1 buffer (50 mM Tris-HCl, pH 8.0, 50 mM NaCl, 7 mM β-mercaptoethanol), oligonucleotide (50 nM substrate of TDP1), testable inhibitor and purified TDP1 (1.5 nM). The reactions were incubated at a constant temperature of 26 °C in a POLARstar OPTIMA fluorimeter, BMG LABTECH, GmbH, to measure fluorescence every 55 s (Ex485/Em520 nm) during the linear phase (here data from 0 to 8 min). The average values of half maximal inhibitory concentration (IC_50_) were determined using an eleven-point concentration response curve in three independent experiments and calculated using MARS Data Analysis 2.0 (BMG LABTECH).

### 2.5. Gel-based TDP1 inhibition assay

TDP1 gel-based assays were performed using 50 nM substrate incubated with 1.5 nM recombinant human TDP1 in the absence or presence of inhibitor for 20 min at 26 °C in a buffer containing 50 mM Tris-HCl, pH 8.0, 50 mM NaCl, 7 mM β-mercaptoethanol. Reactions were terminated by the addition of gel loading buffer (TBE, 10% formamide, 7 M carbamide, 0.1% xylene cyanol, and 0.1% bromophenol blue, 20 mM EDTA). The samples were heated before loading at 90 °C for 7 min. The products were analysed by electrophoresis in a 20% denaturing PAGE with 7 M carbamide at a ratio of acrylamide to bisacrylamide 19:1. Gel images were scanned using a Typhoon FLA 9500 (GE Healthcare) and calculated using a QuantityOne 4.6.7 software.

### 2.6. Fluorescence anisotropy assay

Fluorescence anisotropy was measured by using a fluorescent single-strand DNA-oligonucleotide (3 nM) without quencher at the 3′-end in the presence of the inhibitor in different concentrations (Fluorescein control, C_Flu_ = 10^−9 ^M). Reaction mixtures (200 µl) contained TDP1 buffer (50 mM Tris-HCl, pH 8.0, 50 mM NaCl, 7 mM β-mercaptoethanol), a single-strand DNA-oligonucleotide without quencher (3 nM substrate of TDP1), a testable inhibitor and the purified TDP1 (50 nM). The reactions were incubated at a constant temperature of 26 °C in a POLARstar OPTIMA fluorimeter, BMG LABTECH, GmbH, to measure fluorescence anisotropy every 55 s (Ex482/Em530 nm) during the linear phase (from 0 to 8 min) in two independent experiments.

Fluorescence anisotropy (r) was calculated by using the Equation (1):

(1) $$\text{r }=\;(I_{II}–I_{⊥})/(I_{II}+\text{ 2}I_{⊥})$$

*I_II_* and *I*_⊥_ are the intensities of the fluorescence components polarised parallel and perpendicular to the source of light emission.

The dependence of the anisotropy on the concentration of the inhibitor was determined using a six-point response curve in two independent experiments and calculated using MARS Data Analysis 2.0 (BMG LABTECH).

### 2.7. Steady-state kinetic analysis of enzymatic reaction

To determine the kinetic parameters: apparent maximum rate of enzymatic reaction (V_max_), Michaelis constant (K_M_), constant of inhibition (K_I_), and possible inhibition mechanism, steady-state kinetic experiments were carried out at five fixed concentrations of substrate with variation of inhibitor concentrations. The standard reaction mixtures (200 μl) contained 50 nM, 100 nM, 200 nM, 500 nM or 1000 nM substrate, testable inhibitor, 1.5 nM recombinant human TDP1 and reaction buffer components (50 mM Tris-HCl, pH 8.0, 50 mM NaCl, 7 mM β-mercaptoethanol). After addition of the enzyme, the reaction mixtures were incubated at a constant temperature of 26 °C and measured in a POLARstar OPTIMA fluorimeter, BMG LABTECH, GmbH, to measure fluorescence every 55 s (Ex485/Em520 nm) during the linear phase (data from 0 to 8 min). The initial data (kinetic curves) were obtained in three independent experiments and statistically processed in software OriginPro 8.6.0.

### 2.8. Cell culture assays

Tumour cell line A549 (adenocarcinomic human alveolar basal epithelial cells) and noncancerous cell line WI-38 (fibroblasts derived from lung tissue of a 3 months gestation female fetus) were used in cell-based experiments to determine the cytotoxicity of inhibitors and their effect on antitumour drug topotecan using standard MTT test^48^. Cells were incubated in Iscove’s Modified Dulbecco’s Medium (IMDM) supplemented with 10% fetal bovine serum and 40 µg/ml gentamicin, 10 U/ml penicillin, 10 mg/ml streptomycin, 0.25 µg/ml amphotericin at 37 °C and 5% CO_2_ for 24 h in a CO_2_ incubator; the optical density of the final solution was measured in the wells of the microplate at 570 nm and 620 nm wavelengths using a MultiScan FC (Thermo Scientific) microplate reader.

To study the effect of inhibitors on the cytotoxic effect of a well-known antitumour drug, the topotecan of the manufacturer “ACTAVIS GROUP PTC ehf.” was used. First of all, the 50% cytotoxic concentration (CC_50_) for topotecan and each inhibitor were determined to produce a defined single-agent effect. Second, three independent MTT-tests were performed with each inhibitor in combination with topotecan. Then, the synergistic coefficient (SC) was calculated according to the following formula (2)^49^:

(2) $$\text{SC}=\;\frac{{\text{CC}}_{50}\;\text{tpc},(+I)}{{\text{CC}}_{50}\;\text{tpc}}+\frac{{\text{CC}}_{50}\;I,\;(+\text{tpc})}{{\text{CC}}_{50}\;I},$$

CC_50 tpc,(+I)_ and CC_50 I,(+tpc)_ are the concentrations of topotecan and inhibitor used in combination to achieve 50% drug effect. CC_50 tpc_ and CC_50 I_ are the concentrations for single agents to achieve the same effect. SC values of less than, equal to, or more than 1 indicates synergy, additivity, and antagonism, respectively. The results were calculated and statistically processed in Microsoft Excel.

## 3. Results and discussion

### 3.1. Inhibition of human recombinant TDP1 by disaccharide nucleosides analogues using single-stranded fluorescent biosensor

It is known that the main function of TDP1 is specific hydrolysis of the covalent bond between DNA 3′-phosphate and a tyrosine residue of Topoisomerase 1 (Top1)^4–8^. Moreover, TDP1 is capable to catalyse cleavage of variety of other moieties, which block the 3′-end of the DNA including physiological substrates (3′-phosphoglycolates^50^^,^^51^, 3′-abasic sites^45^^,^^52^, 3′-nucleosides, and 3′-ribonucleosides^53^) as well as numerous synthetic substrates attached to DNA 3′-phosphate such as biotin and different fluorophores^54–57^.

To discover inhibitors of TDP1 we recently developed original single-stranded and hairpin fluorophore–quencher coupled DNA-biosensors for real-time measurement of TDP1 cleavage activity^46^. The single-stranded substrate is a 16-mer oligonucleotide containing both 5′-FAM fluorophore donor and a quenching 3′-BHQ moiety (Figure 1(A)). When the quencher is removed by TDP1, the fluorescence intensity increases and can be measured by fluorimeter (TDP1 catalysed reaction on biosensor is shown in Figure 3(A). Fluorescence intensity declines in the presence of inhibitor, the resulting curves of the dependence of TDP1 residual activity as a function of the concentration of inhibitors were obtained using 4-parameter logistic regression fit, which was used to calculate IC_50_ values^47^^,^^58^.

For primary screening, the effect of a large group of disaccharide nucleosides and their hydrophobic derivatives on human recombinant TDP1 activity has been evaluated by fluorescent method on a single-stranded substrate described above. The results for calculated values of IC_50_ for these compounds are presented in Table 1.

All the studied compounds were divided into three classes: (1) compounds containing (1′→2′)-glycosidic bond between two ribofuranosyl moieties (2′-*O*-pentafuranosylnucleosides); (2) compounds containing β(1′→3′)-glycosidic bond (3′-*O*-β-D-ribofuranosylnucleosides) and also (3) compounds with β(1′→5′)-glycosidic bond (5′-*O*-β-D- ribofuranosylnucleosides).

#### 3.1.1. 2′-O-Pentafuranosylnucleosides

The most effective inhibitor of TDP1 activity (**16,** IC_50_ = 0.4 ± 0.1 µM) among nucleoside derivatives presented in Table 1 is referred to the first class of compounds and represents 2′-*O*-β-D-ribofuranosylnucleoside analogue possessing 2′,3′,5′-*O*-benzoyl groups at the second ribofuranosyl moiety, 3′,5′-di-*O*-tetraisopropyldisiloxane (TIPDS) protective group and 4-benzoylcytosine. The absence of TIPDS in compound **13** has led to the less effective inhibition of TDP1 activity with 6.5 times higher IC_50_ value (2.6 ± 0.2 µM) for compound **13** in comparison with **16**. The same is typical for compounds **14** and **15** with arabinofuranosyl moiety at 2′-position of nucleoside: inhibitory activity of disaccharide with TIPDS is higher than activity of compound without TIPDS. The same modifications of molecular skeleton (TIPDS and benzoyl groups) in disaccharide nucleosides containing other bases also led to TDP1 inhibition in low micro molar range. For disaccharide analogue of **16** containing *N*^6^-benzoyladenine instead of *N*^4^-benzoylcytosine (**4**) IC_50_ value is 1.3 ± 0.2 µM. IC_50_ values for the similar compounds containing thymine (**7**), guanine (**11**), uracil (**21**) are roughly the same.

Compound **6** based on thymine, possessing monophosphate residue as a sodium salt at 5′-position, is characterised by higher IC_50_ value (18.5 ± 0.8 µM). Despite this fact compound **6** seems to be promising for further investigations because of its high water solubility, unlike other TDP1 inhibitors that are soluble in DMSO. High water solubility significantly simplifies the studies directed on a drug development on the basis of nucleosidic TDP1 inhibitors. Compounds **3** and **9** containing the same modification as compound **6** are also water-soluble, but compound **3** containing adenine demonstrates a low inhibitory effect and compound **9** containing guanine base was not active at all. Hence, the structure of heterocyclic base is essential for the inhibitory activity.

#### 3.1.2. 3'-O-β-D-Ribofuranosyl-2′-deoxynucleosides

The presence of bulky groups at both ribofuranosyl moieties results in increasing of the inhibitory activity for this class similar to the class described above. Appending of *tert*-butyldiphenylsilyl (TBDPS) at 5′–position of nucleoside increases the efficiency of TDP1 inhibition about 1.6 times for the cytosine derivative (IC_50_ = 0.9 ± 0.1 μM for **37** compared to IC_50_ = 1.5 ± 0.2 μM for **36**) and about 3.5 times for the uracil derivative (IC_50_ = 0.8 ± 0.1 μM for **42** compared to IC_50_ = 2.8 ± 0.2 μM for **41**).

Noteworthy, the modification of thymine disaccharide nucleoside belonging to the current class of compounds with phosphate group as an ammonium salt at 5′-position of the first ribose moiety (**26**) does not lead to the noticeable inhibitory effect. The presence of phosphate group at 5′-position of the second ribose moiety (**25**) also does not lead to the noticeable inhibitory effect. These data suggest that only simultaneous presence of 5′-phosphate group and β(1′→2′)-glycosidic bond between ribofuranosyl residues leads to the inhibitory effect on TDP1 activity as in case of compounds **3** and **6**.

We did not observe any inhibitory effect when the second ribofuranosyl moiety was changed on fully acetylated β-D-ribopyranosyl moiety (**28**) or β-D-ribopyranosyl moiety possessing free hydroxyl groups (**27** and **40**). Oxidation of *cis*-diol system of the second ribofuranosyl moiety in **24** and **31** to give compounds **29** and **30** respectively did not lead to the noticeable inhibitory activity.

It is interesting to note that the compounds **24** and **29** are the inhibitors of other key DNA repair enzyme – PARP1 in the micromolar range (IC_50_ = 38 ± 4 μM for compound **24** and IC_50_ = 25 ± 3 μM for compound **29**)^28^. Recently, it was determined that interaction of Tdp1 with damaged DNA depends on PARP1^59^. The protein–protein interaction of PARP1 with TDP1 were also detected and estimated quantitatively using fluorescent titration techniques^60^^,^^61^.

PARP1 regulates TDP1 repair activity in cells using the N-terminal domain of TDP1 which directly binds the C-terminal domain of PARP1 resulted in PARylation of TDP1 by PARP1^59^. This process plays a critical role in choosing the repair pathway for Top1 cleavage complexes (it can be TDP1-repair pathway^8^^,^^12^ or endonuclease cleavage pathway^13^^,^^14^). Thus, combination of PARP1 and Top1 inhibitors should be most beneficial in endonuclease-deficient cancer cells, such as ERCC1-deficient^62^ or Mre11-deficient tumours^63^. Therefore, search and development of such double PARP1-TDP1 inhibitors appear to be a promising task for future investigations.

The presence of two 2′,3′,5′–tri-*O*-benzoyl-β-D-ribofuranosyl moieties attached to thymidine at 3′- and 5′-positions has led to the quite strong inhibitory effect (compound **33** with IC_50_ = 1.0 ± 0.1 μM) despite this inhibitor has no silyl groups.

#### 3.1.3. 5′-O-β-D-Ribofuranosylnucleosides

Compounds containing 5′-1′-glycosidic bond between two ribofuranosyl residues did not inhibit TDP1 activity except non-modified uracil (**47**) and guanine (**45**) nucleosides. Modification of uracil with iodine or fluorine groups at position 5 led to the lack of inhibitory effect. Nevertheless, further enhancement of this nucleosidic library with new derivatives may probably increase their inhibitory effect.

Thus, the screening conducted on a series of disaccharide nucleosides has shown that enzymatic activity of TDP1 is not inhibited by non-modified disaccharide nucleosides containing adenine (**1**, **22, 43**), thymine (**5**, **24, 44**), guanine (**8** and **34**), cytosine (**12**, **35, 46**), and uracil (**17** and **38**) as heterocyclic bases; disaccharide nucleosides based on guanine (**45**) and uracil (**47**) with β(1′→5′)-glycosidic bond display weak inhibitory effect on TDP1 activity.

As shown in Table 1, the most effective inhibitors of TDP1 contain the modification of 2′,3′,5′-hydroxyl groups of the second ribofuranosyl residue with benzoyl groups (**4, 7, 10, 11, 13, 14, 15, 16, 20, 21, 33, 36, 37, 41, 42**). The inhibitory effect increases after appending of bulky silyl groups into the first ribofuranosyl moiety at 3′,5′-positions in the case of 2′-*O*-β-D-ribofuranosylnucleosides **(11, 15, 16, 21)** or 5′-position in the case of 3′-*O*-β-D-ribofuranosyl-2′-deoxynucleosides **(37, 42).** The presence of such groups allows both introducing of additional functionalities into nucleosidic structure and increasing of hydrophobicity that may improve the penetrability of disaccharide nucleosides through cellular membranes.

The obtained data allowed the identification of disaccharide nucleosides as a novel class of TDP1 inhibitors, which has not been previously described. The most efficient TDP1 inhibitors manifest IC_50_ values in low-micromolar range. The key structural motif of such compounds is 2′,3′,5′-tri-O-benzoylpentafuranose residue.

### 3.2. The effect of inhibitors on cell growth and viability and their effect in combination with topotecan

Developing enzyme inhibitors as prototypes of drugs for combination therapy requires the evaluation of their impact on cytotoxicity in combination with drug used in clinical practice. It is expected that TDP1 inhibitors will be able to enhance sensitivity of cancer cells to topoisomerase 1 poisons, such as camptothecin and its derivatives (irinotecan, topotecan).

Tumour cell line A-549 (adenocarcinomic human alveolar basal epithelial cells) and noncancerous cell line WI-38 (fibroblasts derived from lung tissue of a 3 months gestation female fetus) were used for MTT-test. We chose the most effective TDP1 inhibitors (**4, 6, 7, 10, 11, 13, 14, 15, 16, 21, 33, 36, 37, 41, 42**) based on fluorescence screening (IC_50_ values in Table 1). For these 15 compounds, MTT-test was performed.

The 50% cytotoxic concentration (CC_50_) was defined as a concentration required to reduce a number of viable cells by 50% compared to that for the untreated controls containing 1% of DMSO (for **4, 7, 10, 11, 13, 14, 15, 16, 21, 33, 36, 37, 41, 42** inhibitors) and without DMSO (for the water-soluble inhibitor **6**). Determined CC_50_ values for most of these compounds were higher than 100 μM for both cell lines as indicated in Table 2, thus the inhibitors demonstrate quite low own cytotoxicity. This is of great importance for their application in drug combination therapy as an anti-cancer treatment since it is critical that the use of new compounds does not lead to additional toxic load.

**Table 2.** Cytotoxicity of inhibitors, topotecan and their combination for A-549 and WI-38 cell lines.

I: inhibitor; tpc: topotecan; SC: synergistic coefficient. The inhibitor 6 was dissolved in water. Other inhibitors were dissolved in DMSO. Red colour indicates inhibitors that enhance cytotoxicity of topotecan for A-549 cell line; blue colour: for the WI-38 line.

The CC_50_ values for topotecan in DMSO and in water (for inhibitor **6**) also are shown in Table 2. These values were significantly higher for A-549 cell line, which is probably because the non-cancerous WI-38 cell line is more sensitive to the treatment with topotecan. Reducing a topotecan dosage for A-549 leads to a considerable decrease of topotecan effect for this cell line and, as a consequence, we did not determine any effect of the inhibitors. At the same time increasing a topotecan dosage for WI-38 cell line will be followed by significant cell death and again we did not determine any effect of the inhibitors. Thus, alignment of topotecan concentration for both cell lines is impossible, because topotecan influence on the A-549 and WI-38 cell survival is in different diapasons of concentrations.

Then the influence of inhibitors to cytotoxic effect of anti-cancer drug topotecan was estimated for the same cell lines. The used inhibitor’s concentrations were non-toxic for cells (much less than CC_50_ values) but were not lower than the corresponding IC_50_ values. Then, the synergistic coefficients were calculated (Table 2) as was described in 2.8. Determination of synergistic coefficient evaluates the nature of interaction of two agents, and numerical value of SC also provides a quantitative measure of the extent of drug interaction^49^.

**Figure 2.**
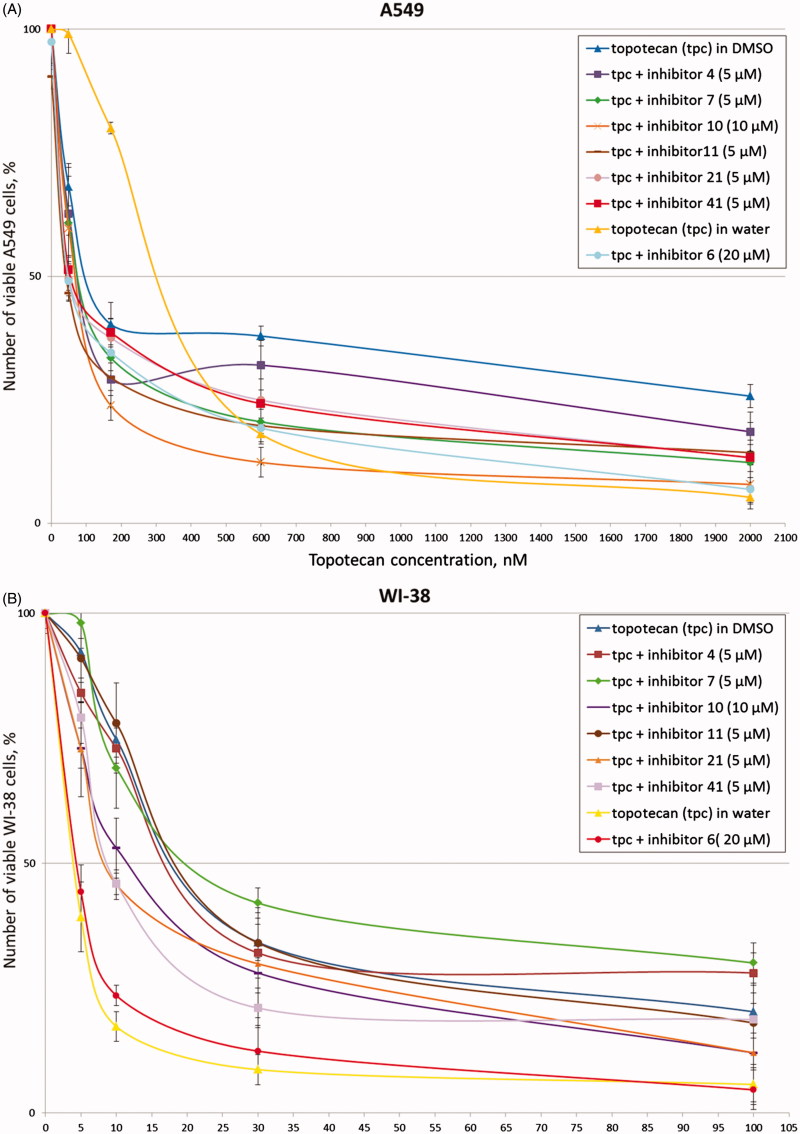
Topotecan dose-dependent action of the disaccharide nucleosides on A-549 (A) and WI-38 (B) viability according to the MTT-assay. Average data with error bars from three independent experiments.

After addition of the inhibitors in combination with topotecan to A-549 cells, we observed significant increase of cytotoxic effect of topotecan for seven inhibitors (**4, 6, 7, 10, 11, 21, 41**). Typical experimental curves for these inhibitors are shown in Figure 2(A). The synergistic coefficients were less than or equal to 0.7 for these compounds (Table 2). Synergistic effect proved to be the largest for inhibitors **6** and **11** (SC values are 0.4). It is to be noted that for inhibitor **6** we used water-soluble topotecan as a control because this inhibitor is water-soluble. At the same time only three inhibitors (**10, 21, 41**) demonstrated synergy with topotecan for non-cancerous cell line WI-38 (Table 2, Figure 2(B)). Obtained SC values were lower than corresponding values for A-549 cell line. Thus, for further research we selected seven (**4, 6, 7, 10, 11, 21, 41**) the most effective inhibitors that can increase cytotoxicity of topotecan.

### 3.3. Investigation of inhibitory activity using a gel-based assay

**Figure 3.**
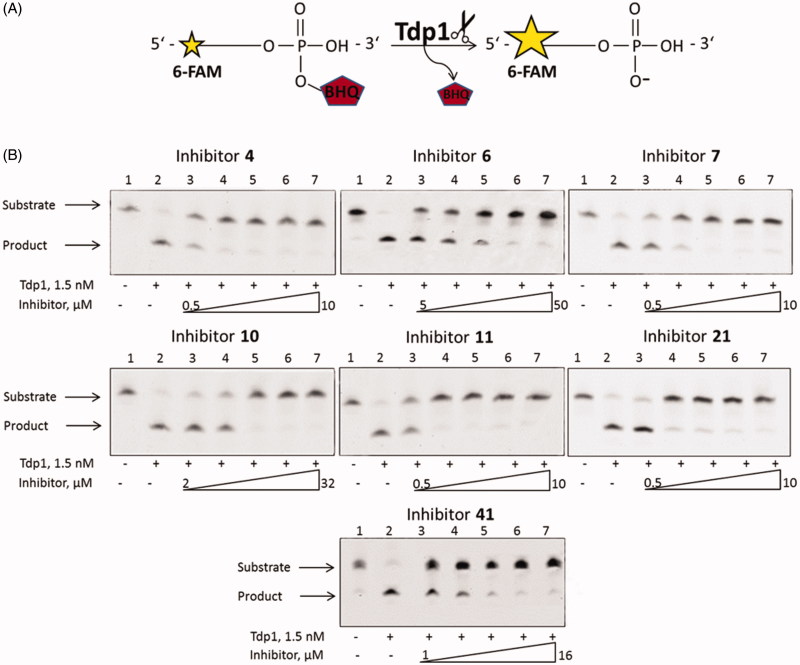
(A) TDP1 reaction scheme with single-stranded biosensor. (B) Gel pictures of the TDP1 reaction products. The arrows indicate the positions of the initial substrate and the reaction product. Concentration of TDP1 was 1.5 nM, concentration of single-stranded substrate was 50 nM, and reaction time was 20 min.

To confirm the data obtained by the fluorescence method, we performed an electrophoretic separation in polyacrylamide gel of the reaction products catalysed by TDP1 (Figure 3(A)) using a single-stranded oligonucleotide described in 2.3. Representative gels for the most effective 7 inhibitors are shown in Figure 3(B).

According to the gel pictures the quencher is cleaved by TDP1 in the absence of an inhibitor (Figure 3(B) lanes 2). As the inhibitor concentration increases, the amount of the product decreases (Figure 3(B) lanes 3–7), leading to an increase in the inhibition efficiency. The IC_50_ values obtained by the fluorescent method (Table 1), fall within the range of inhibitor concentrations in Figure 3(B). Thus, the data obtained by this method correlates with the data obtained using the fluorescent assay (Table 3).

**Figure 4.**
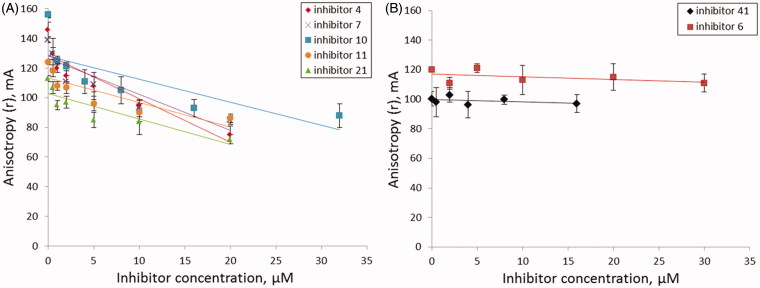
The dependence of fluorescence anisotropy on inhibitor concentration (A) for inhibitors **4**, **7**, **10**, **11**, **21**; (B) for inhibitors **6**, **41**. Average data with error bars from two independent experiments.

**Figure 5.**
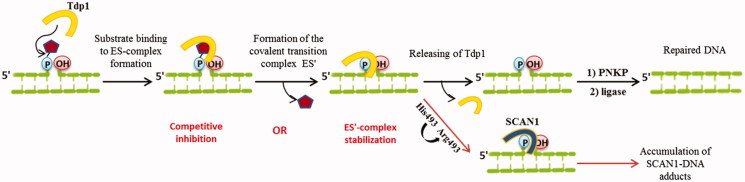
TDP1 action and possible inhibition strategies. Yellow shape represents TDP1; pentagon represents Top1 residue in vivo or BHQ in this study.

**Figure 6.**
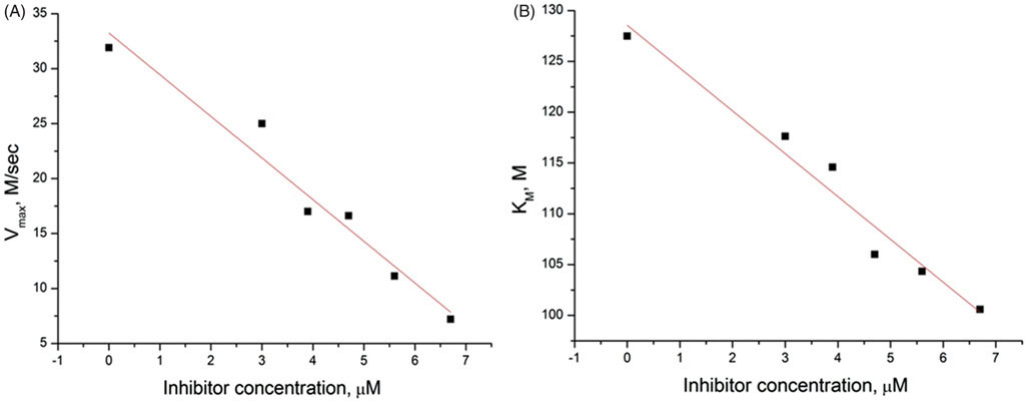
(A) Dependence of V_max_ on the concentration of inhibitor **41**. (B) Dependence of K_M_ on the concentration of inhibitor **41**.

**Table 3.** IC_50_ values for selected inhibitors obtained on single-stranded and hairpin substrates.

### 3.4. Determination of IC_50_ values on a hairpin substrate by a fluorescent method

For seven the most effective inhibitors IC_50_ values were determined using a short hairpin oligonucleotide with a 1,10-decanediol loop, a 5′-fluorophore, and a 3′-quencher attached via a non-digestible 3′-phosphate analogue - tetramethyl phosphoryl guanidine (TMG) group (Figure 1(B)). Such structure of the oligonucleotide allowed to improve sensitivity and selectivity of the assay^39^. In this case, cleavage catalysed by TDP1 occurs in the middle of the chain with the removal of fluorophore group because attached TMG group is resistant to 3′-phosphodiesterase cleavage by TDP1.

Similarly to the experiments with a single-stranded substrate (section 3.1), fluorescence intensity decrease curves were obtained for the hairpin substrate in the presence of tested inhibitors, and IC_50_ values were calculated, which practically did not quite differ from the values obtained on a single-stranded substrate (within the measurement accuracy) (Table 3). Thus, there is an agreement between IC_50_ values determined independently using two types of DNA substrates for the selected inhibitors.

### 3.5. Influence of disaccharide nucleosides on SCAN1 activity

It is known that the inherited disorder, spinocerebellar ataxia with axonal neuropathy (SCAN1), is caused by a H493R mutation in *TDP1* gen^64^. The His493 residue participates in the second step of TDP1 reaction and mediates the dissociation of the enzyme from the transition complex^12^^,^^65^. In the case of H493R mutation, this step of catalysis is not realised and the mutated TDP1 (SCAN1) remains bound to DNA, thereby causing a cumulative effect of the accumulation of DNA-SCAN1 adducts in cells (Figure 5)^17^. This is also confirmed by the data that in patients with SCAN1 this phenotype does not appear until the second decade of life^64^. Therefore, the study of the inhibitory activity of compounds on the mutant form, allows making assumptions about which catalytic step the inhibitor acts on, and also it is an important component of further research aimed at developing of effective SCAN1 inhibitors.

We tested seven the most effective TDP1 inhibitors (**4, 6, 7, 10, 11, 21, 41**) as potential inhibitors of SCAN1. The IC_50_ values obtained by the fluorescence real-time assay on a single-stranded oligonucleotide (section 3.1) were higher than 100 μM. Hence, these tested compounds are not effective inhibitors of SCAN1 and they are selective for TDP1 in this context.

The fact that selected compounds inhibit only TDP1 and do not inhibit mutant form of TDP1 suggests that they apparently affect the second step of enzymatic reaction – a release of the enzyme from the transition complex because this step is absent in the case of SCAN1.

### 3.6. Measurement of fluorescence anisotropy in the presence of inhibitors

To obtain some information about interaction of disaccharide nucleosides with TDP1, anisotropy of the fluorescent DNA substrate was measured. We used single-stranded oligonucleotide without a quencher at the 3′-end in the presence of testable inhibitors at different concentrations. Thus, this oligonucleotide is involved in a reversible binding step of the TDP1 reaction, but the catalytic step of 3'-quencher group removal does not occur.

When TDP1 is added to the DNA substrate, the fluorophore mobility decreases and, as a consequence, the anisotropy level increases, reflecting the degree of complexation of the enzyme with the DNA substrate^66^. Addition of an inhibitor to this TDP1-substrate mixture resulted in a change in the anisotropy level depending on the mechanism by which the inhibitor acts. Among seven TDP1 inhibitors (**4, 6, 7, 10, 11, 21, 41**), a significant decrease in the anisotropy dependent of inhibitor concentration was observed for five of them (**4, 7, 10, 11, 21**); for two of them (**6** and **41**) the anisotropy remained unchanged (Figure 4).

According to Figure 4, B anisotropy level is constant after addition of inhibitors **6** and **41** which suggests that fluorophore mobility remains constant. This may indicate that DNA substrate remains bound in the active site of TDP1 and inhibitors **6** and **41** do not belong to competitive inhibitors. Hence, for these two inhibitors, the type of inhibition may be either non-competitive (the inhibitor is able to bind both the enzyme-substrate complex and free enzyme) or uncompetitive (the inhibitor binds only the enzyme-substrate complex). However, it is impossible to distinguish these two types, because under our conditions, with an excess of the enzyme, the contribution from the binding of a noncompetitive inhibitor is invisible. Moreover, the observed decrease in the anisotropy level for the inhibitors **4, 7, 10, 11** and **21** can be attributed not only to the competitive but also to the mixed type of inhibition. Therefore, in order to clarify the type of inhibition and to receive more detailed information, it is necessary to determine kinetic parameters of the enzymatic reaction in the presence of the inhibitors.

### 3.7. Enzyme kinetic parameters and inhibition mechanism

The presence of an inhibitor in the reaction mixture affects the kinetic parameters of the enzymatic reaction. The determination of the enzyme behavior in the presence of inhibitors and the estimation of kinetic parameters in this process can reveal a significant insight into the mechanisms of inhibition.

According to the Michaelis–Menten model^67^, the substrate (S) binds to the enzyme (E) to form the complex (ES), which is then converted to the product (P) and the free enzyme. However, it is known that TDP1 hydrolyses DNA-Top1 adducts *via* two coordinated S_N2_ nucleophilic attacks with the formation of a transition covalent intermediate (Figure 5)^12^^,^^68^. In this way, TDP1 reaction has more complex kinetic scheme, supplemented by one more step - formation of the covalent transition complex (ES′). Thus, there are two potential strategies for inhibiting TDP1 activity: the inhibition of the first step of TDP1 catalysis, i.e. binding of the enzyme to its substrate (the nucleophilic attack of His263) or blocking the other step, i.e. release of the enzyme from this transition complex (nucleophilic attack by a water molecule activated by His493) preventing the formation of final 3′-phosphate product and free Tdp1 (this is also observed for catalysis by SCAN1 mutation form) (Figure 5)^12^^,^^68^. This model of Tdp1 action is reduced to the general Michaelis–Menten model under steady-state conditions.

The dependence of the reaction rate on the substrate concentration and dependence of V_max_ and K_M_ on the concentration of the inhibitor were obtained for the most effective inhibitors under steady-state conditions. For instance, corresponding curves for inhibitor **41** are presented in Figure 6. V_max_ and K_M_ decrease proportionally (Figure 6(A,B)), which corresponds to the uncompetitive type of inhibition, according to the general equation of the rate of product formation in the Michaelis–Menten kinetics^67^.

This assumption is confirmed by the combined analysis of plots, obtained by linearizing the kinetic curves using Lineweaver–Burk^69^ and Eadie–Hofstee models^70^^,^^71^ (see the supplemental material).

Values of the inhibition constant (K_I_) were determined using Cornish–Bowden method^72^. Similar experiments were performed for all seven tested inhibitors on two types of DNA substrates, and the results are shown in Table 4.

**Table 4.** Type of inhibition and values of inhibition constant (K_I_) for selected inhibitors.

Compound number	Single-stranded substrate		Double-stranded substrate	
	Inhibition constant, K_I_, µM	Type of inhibition	Inhibition constant, K_I_, µM	Type of inhibition
**4**	0.3 ± 0.1	Mixed	0.2 ± 0.1	Mixed
**6**	7.9 ± 0.8	Noncompetitive	8.8 ± 0.9	Noncompetitive
**7**	0.9 ± 0.3	Mixed	0.3 ± 0.1	Mixed
**10**	1.7 ± 0.4	Mixed	3.1 ± 0.3	Mixed
**11**	0.2 ± 0.1	Mixed	0.3 ± 0.2	Mixed
**21**	0.3 ± 0.2	Mixed	0.4 ± 0.3	Mixed
**41**	0.6 ± 0.3	Uncompetitive	2.1 ± 0.5	Uncompetitive

According to Table 4, among all seven inhibitors only inhibitor **41** has demonstrated uncompetitive type of inhibition, when the inhibitor binds enzyme-substrate complex (ES) but does not bind free enzyme in the absence of a substrate. It is possible that position of the bond between ribofuranose moieties exerts key influence on the type of inhibition for this compound because only inhibitor **41** refers to 3′-O-β-D-ribofuranosyl-2′-deoxynucleosides with the β(1′→3′)-glycosidic bond between ribofuranoses.

Inhibitor **6** refers to non-competitive inhibitors, i.e. it can decelerate catalysis by binding either to the enzyme-substrate complex or to free enzyme. Seemingly, ability to bind also free enzyme is associated with the fact that inhibitor **6** is negatively charged (contains a positive counter-ion) and has no bulky substituents in its composition in contrast to other inhibitors. This can promote its better binding in the relatively narrow and positively charged cleft located on one side of the catalytic pocket of TDP1^70^. The electrostatic binding of the enzyme with the negatively charged phosphate backbone of single-stranded DNA of the substrate occurs in this cleft^73^^,^^74^.

Most of the inhibitors investigated in this work predominantly show a mixed type of inhibition, which is often observed for complex kinetic schemes and two-substrate reactions. The action of mixed inhibitor is similar to a non-competitive inhibitor, but it affects not only V_max_ but also K_M_. During a process of inhibition conformational changes in the enzyme structure occur. As a result, enzyme loses affinity to the substrate, and inhibition of the enzymatic reaction is observed. As it was described above, catalytic pocket of TDP1 has a relatively narrow cleft (responsible for DNA moiety binding), but also it has the other side of the catalytic pocket in a relatively large, more open cleft that contains a mixed charge distribution (there is a fragment of the Top1 located)^73–75^. It is possible, that these mixed inhibitors block this cleft of TDP1 because all of them have bulky modifications such as 2′,3′,5′-tri-*O*-benzoylpentafuranose residues, which can be retained in this site due to polar interactions involving 5′-phosphate groups of three phosphotyrosyl linking nucleotides and the amino acid residues Ser400, Ser403, Lys469, Ser518, Lys519 and Ala520^74–77^. Moreover, most of these inhibitors (**4, 7, 11, 21**) have bulky silyl groups in their structures, which may play an essential role in hydrophobic interactions inside the TDP1 cleavage site^65^^,^^75^^,^^77^. Mixed inhibitor **10** does not have any hydrophobic silyl modifications, which is reflected in a higher value of the inhibition constant characterising stability of the enzyme-inhibitor complex. Other mixed inhibitors have particularly the same relatively small values of K_I_. This assumption is consistent with the fact that the absence of any bulky groups leads to increasing of the K_I_ value like for inhibitor **6.** Thus, in this work, it was determined that all the studied disaccharide nucleosides have not fully competitive mechanism of inhibition that is of great importance for further development of sensitisers to topotecan and other clinically used Top1 inhibitors.

## 4. Conclusions

Disaccharide nucleosides is a group of natural compounds forming poly(ADP-ribose) and found in tRNA, antibiotics, and other physiologically active compounds^22–25^. Here, we tested for the first time a large group of disaccharide nucleosides and their hydrophobic derivatives as potential inhibitors of human recombinant TDP1 using real-time fluorescence assay. The screening revealed several effective inhibitors of TDP1, including water-soluble inhibitors. The obtained IC_50_ values were measured using two types of DNA-oligonucleotides and lay within the low micro molar range. These results allow identifying disaccharide nucleosides as a new class of TDP1 inhibitors, which was not previously reported.

It was shown, that the key structural motif which is necessary for high inhibitory activity is 2′,3′,5′-tri-O-benzoylpentafuranose residue. This motif appears to be involved in interactions with TDP1 residues. An important contribution to the inhibitory activity of the compounds is also made by the presence of bulky silyl groups in the structures, which are responsible for hydrophobic interactions inside the TDP1 cleavage site. Further investigations in this area will be directed on a structural modification of disaccharide nucleosides to improve their water solubility and inhibitory effect.

Moreover, the TDP1 inhibitors described in this study are not effective inhibitors of the mutant form of TDP1 with H493R substitution (SCAN1). It means that these compounds most likely inhibit the step of the enzyme release from the transition complex during the TDP1 catalysis because SCAN1 is not able to carry out this step^64^. It is important to note that despite the role of TDP1 in DNA repair, SCAN1 patients do not have an increased incidence of cancer or other health problems^17^^,^^51^^,^^64^. Therefore, the defect of catalysis, normally performed by TDP1, does not lead to acute cellular toxicity, but it favors more effective chemotherapy based on Top1 poison-mediated DNA damage, that makes this therapeutic target more attractive.

The own cytotoxicity of the inhibitors and their effect on the cytotoxicity of anti-cancer drug topotecan were determined on cancerous and noncancerous cells. Also, it was shown that these disaccharide nucleosides demonstrate quite low own cytotoxicity, which is important for an application of them in combination with toxic Top1 inhibitors. A significant synergistic effect with topotecan is observed in the presence of some inhibitors. This effect is the most pronounced on cancer cells, thus possible therapeutic effect of these inhibitors could enhance the activity of Top1 inhibitors in tumors with disorders in DNA repair process and cell cycle control.

Finally, the present study provides new insights into the mechanism of action of disaccharide nucleosides on TDP1 activity. Most of the studied inhibitors appear to act as mixed inhibitors, this is often observed for complex kinetic schemes and two-substrate reactions. It was shown by a measurement of kinetic parameters and anisotropy of the enzymatic reaction in the presence of inhibitors using two types of DNA-oligonucleotides. It is most likely that the binding of all the studied disaccharide nucleosides does not occur directly in the active site of TDP1. We will extend further investigation of the interaction mechanism of these analogues with TDP1 in the future. Application of these inhibitors as sensitisers in combination therapy will selectively increase the cytotoxic effect of the anti-cancer drugs that will allow to decrease their therapeutic dose, reducing the total toxicity on the organism. Therefore, such not competitive inhibition is preferable in this case. Thus, obtained results and advantageous properties of disaccharide nucleosides suggest that they are novel promising prototypes of medical drugs for further development of tumour sensitisers based on TDP1 inhibition to available antitumour camptothecin-based drugs.

## Supplementary Material

Supplemental Material
