# Supplementary material for: Novel group of tyrosyl-DNA-phosphodiesterase 1 inhibitors based on disaccharide nucleosides as drug prototypes for anti-cancer therapy
Source: J Enzyme Inhib Med Chem. 2018 Sep 7;33(1):1415–29. doi: 10.1080/14756366.2018.1509210 (PMC6136360; doi:10.1080/14756366.2018.1509210)

Partition coefficient (logP)

To estimate the distribution of the studied compounds within the cell partition coefficient (logP) was calculated for all compounds (see Table 1). Partition coefficient (logP) was determined as the ratio of concentrations of a compound in a mixture of two immiscible phases at equilibrium, one of the solvents being water while the second being 1-octanol. Disaccharide nucleosides in which all the hydroxyl groups are blocked demonstrate the highest hydrophobicity with octanol/water partition coefficients being in the range from 10.08 to 13.27. Therefore, they are distributed mainly to hydrophobic areas such as lipid bilayers of cells. Conversely, disaccharide nucleosides possessing free hydroxyl groups and phosphate groups demonstrate the highest hydrophilicity with octanol/water partition coefficients being in the range from -4.26 to -1.67 and may be found primarily in aqueous regions such as cytosol. Partially deblocked disaccharide compounds are characterized by moderate values of logP and hence are amphiphilic. These compounds may be distributed both to lipid bilayers of a cellular membrane and to aqueous media of a cytosol and an organelles, thus having a potency to penetrate through lipophilic membrane and interact with various proteins inside a cell.

Table 1. Structural formulas and inhibitory activities for the investigated disaccharide nucleosides.

Structural formula	Base	Modifications			IC$_{50}$, µM	logP	Compound number
		Base	The first ribofuranosyl moiety	The second ribofuranosyl moiety			
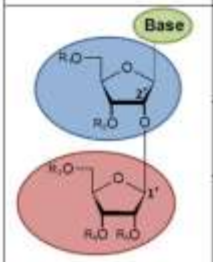	A	-	-	-	>100	-3.23	1
	A	-	-	α-D-ribofuranosyl	>100	-3.23	2
	A	-	-	5'-phosphate (Na$^+$ salt)	40 ± 10	-4.12	3
	A	Ade^{Bz}	3',5'-O-TIPDS	2',3',5'-tri-O-benzoyl	1.3 ± 0.2	11.52	4
	T	-	-	-	>100	-3.16	5
	T	-	5'-phosphate (Na$^+$ salt)	-	18.5 ± 0.8	-3.28	6
	T	-	3',5'-O-TIPDS	2',3',5'-tri-O-benzoyl	1.9 ± 0.3	10.08	7
	G	-	-	-	>100	-3.85	8
	G	-	-	5'-phosphate (Na$^+$ salt)	>100	-4.26	9
	G	Gua^{i-But}	-	2',3',5'-tri-O-benzoyl	5.9 ± 0.7	4.81	10
	G	Gua^{i-But}	3',5'-O-TIPDS	2',3',5'-tri-O-benzoyl	0.7 ± 0.2	9.68	11
3',5'-*O*-TIPDS – tetraisopropyldisiloxane-1,3-diyl	C	-	-	-	>100	-3.94	12
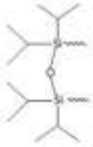	C	Cyt^{Bz}	-	2',3',5'-tri-O-benzoyl	2.6 ± 0.2	5.33	13
	C	Cyt^{Bz}	-	2',3',5'-tri-O-benzoyl-α-L-arabinofuranosyl	1.7 ± 0.4	5.33	14
	C	Cyt^{Bz}	3',5'-O-TIPDS	2',3',5'-tri-O-benzoyl-α-L-arabinofuranosyl	0.6 ± 0.1	11.51	15
	C	Cyt^{Bz}	3',5'-O-TIPDS	2',3',5'-tri-O-benzoyl	0.4 ± 0.1	11.51	16
	U	-	-	-	>100	-3.56	17
	U	-	-	α-D-ribofuranosyl	>100	-3.56	18
	U	5-F-Ura	-	-	>100	-3.36	19
	U	5-F-Ura	-	2',3',5'-tri-O-benzoyl	14 ± 2	4.13	20
	U	-	3',5'-O-TIPDS	2',3',5'-tri-O-benzoyl	0.9 ± 0.1	10.15	21
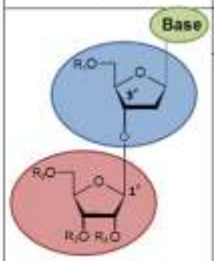	A	-	-	-	>100	-2.33	22
	A	-	α-D-2'-deoxyribofuranosyl	-	>100	-2.33	23
	T	-	-	-	>100	-2.26	24
	T	-	-	5'-phosphate (Na$^+$ salt)	>100	-2.38	25
	T	-	5'-phosphate (NH$_4^+$ salt)	-	>100	-2.38	26
	T	-	-	β-D-ribopyranose	>100	-2.26	27
	T	-	-	β-D-2',3'4'-tri-O-acetylribopyranose	>100	-0.94	28
	T	-	-	2',3' - dialdehyde	>100	-1.96	29
	T	-	α-D-2'-deoxyribofuranosyl	2',3' - dialdehyde	>100	-1.96	30
	T	-	α-D-2'-deoxyribofuranosyl	-	>100	-2.26	31
	T	-	5'-O-β-D-ribofuranosyl	-	>100	-3.4	32
	T	-	5'-O-(2',3',5'-tri-O-benzoyl-β-D-ribofuranosyl)	2',3',5'-tri-O-benzoyl	1.0 ± 0.1	11.57	33
	G	-	-	-	>100	-2.95	34
5'-TBDPS – *tert*-butyldiphenylsilyl	C	-	-	-	>100	-3.04	35
	C	Cyt^{Bz}	-	2',3',5'-tri-O-benzoyl	1.5 ± 0.2	6.23	36
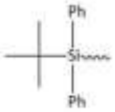	C	Cyt^{Bz}	5'-TBDPS	2',3',5'-tri-O-benzoyl	0.9 ± 0.1	13.27	37
	U	-	-	-	>100	-2.65	38
	U	5-I-Ura	-	-	>100	-1.67	39
	U	5-I-Ura	-	β-D-ribopyranose	>100	-1.67	40
	U	5-I-Ura	-	2',3',5'-tri-O-benzoyl	2.8 ± 0.2	5.82	41
	U	5-I-Ura	5'-TBDPS	2',3',5'-tri-O-benzoyl	0.8 ± 0.1	12.76	42
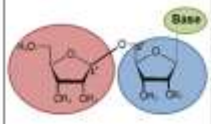	A	-	-	-	>100	-2.33	43
	T	-	-	-	>100	-2.26	44
	G	-	-	-	70 ± 10	-2.95	45
	C	-	-	-	>100	-3.04	46
	U	-	-	-	30 ± 10	-2.65	47
	U	5-I-Ura	-	-	>100	-1.67	48
	U	5-F-Ura	-	-	>100	-2.46	49

1. *R$_x$ – substituting group, in case of "-" R=H;
2. Ade^{Bz} - N^6-Benzoyladenine-9-yl
3. Gua^{i-But} - N^2-Isobutyrylguanine-9-yl
4. Cyt^{Bz} - N^4-Benzoylcytosine-1-yl
5. 5-F-Ura - 5-Fluorouracil-1-yl
6. 5-I-Ura - 5-Iodouracil-1-yl

The examples of typical kinetic plots for selected inhibitors (4, 6, 7, 10, 11, 21, 41) on single-stranded substrate

Inhibitor **4** (mixed) $K_I = 0.3 \pm 0.1$

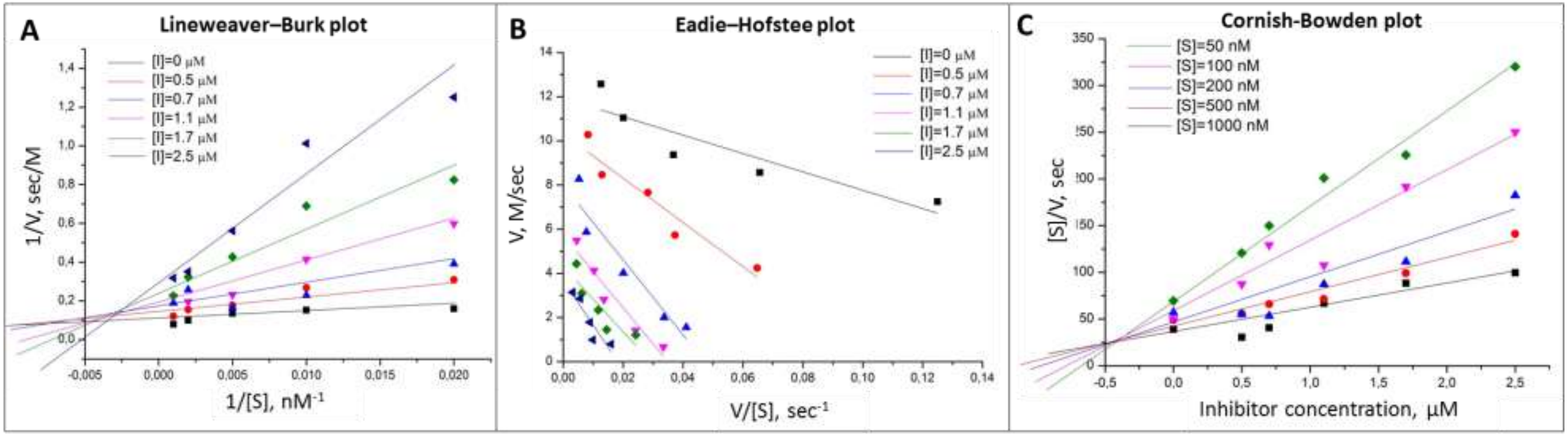

Inhibitor **6** (non-competitive) $K_I = 7.9 \pm 0.8$

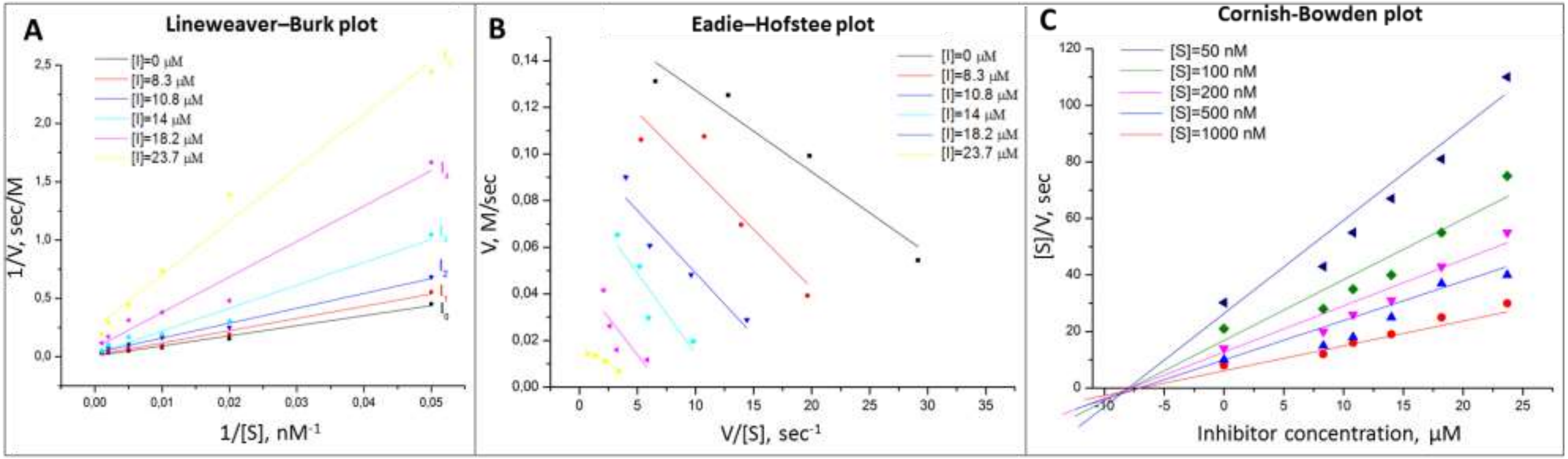

Inhibitor **7** (mixed) $K_I = 0.9 \pm 0.3$

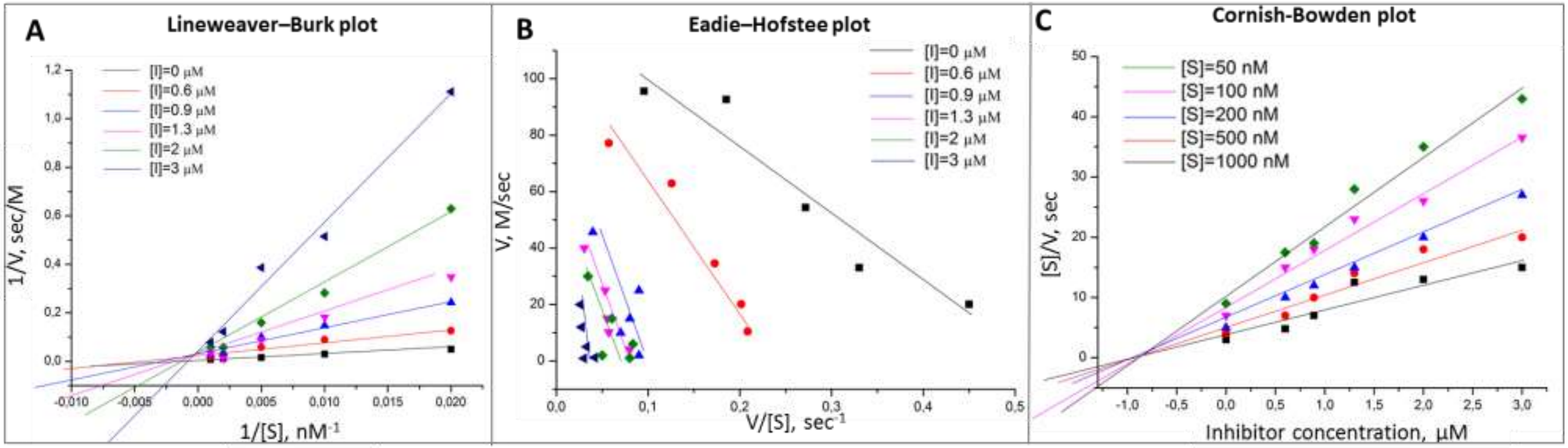

Inhibitor **10** (mixed) $K_I = 1.7 \pm 0.4$

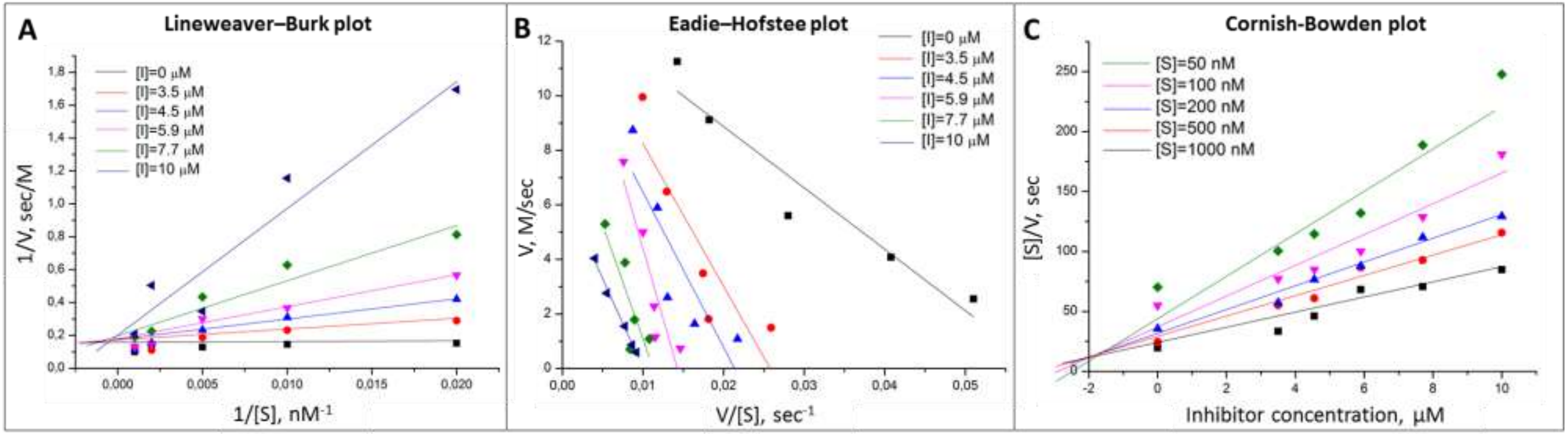

Inhibitor **11** (mixed) $K_I = 0.2 \pm 0.1$

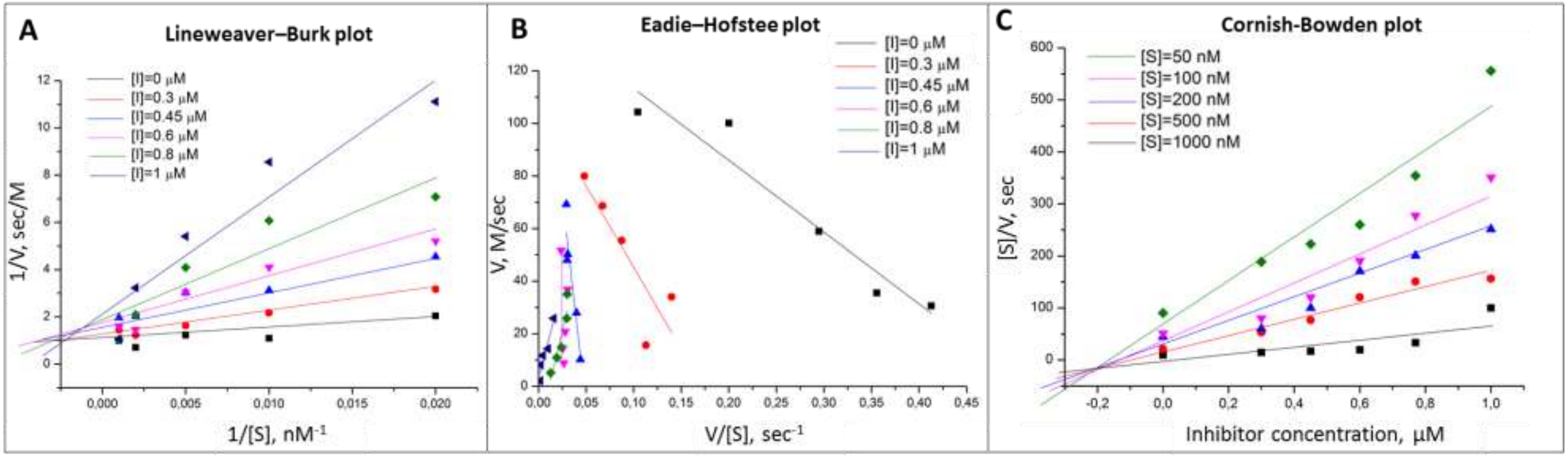

Inhibitor **21** (mixed) $K_I = 0.3 \pm 0.2$

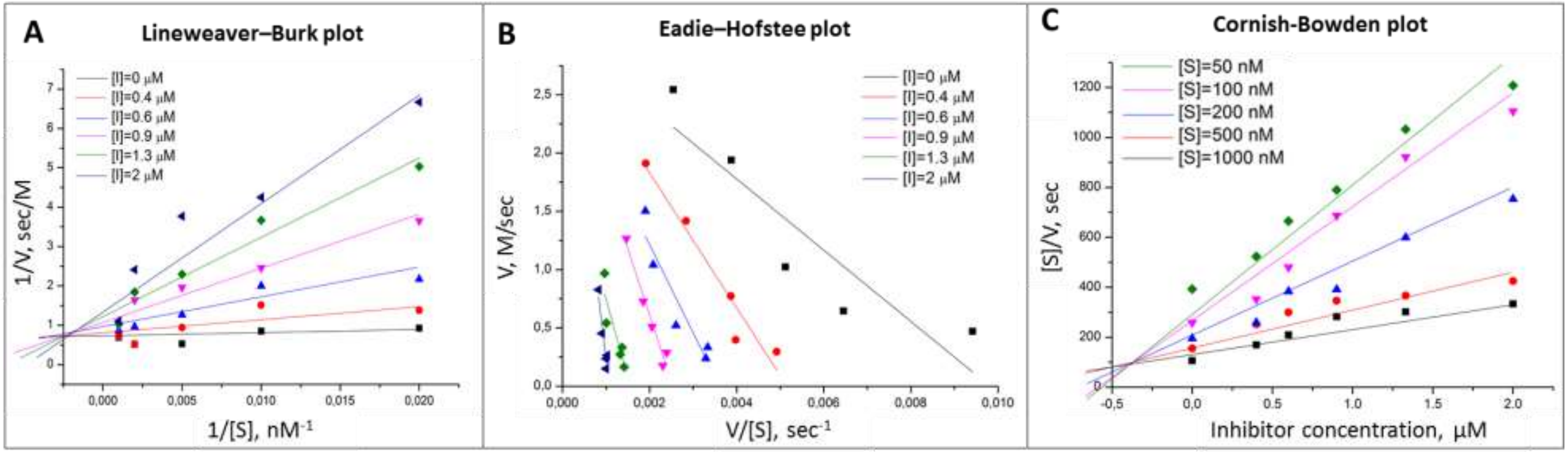

Inhibitor **41** (uncompetitive) $K_I = 0.6 \pm 0.3$

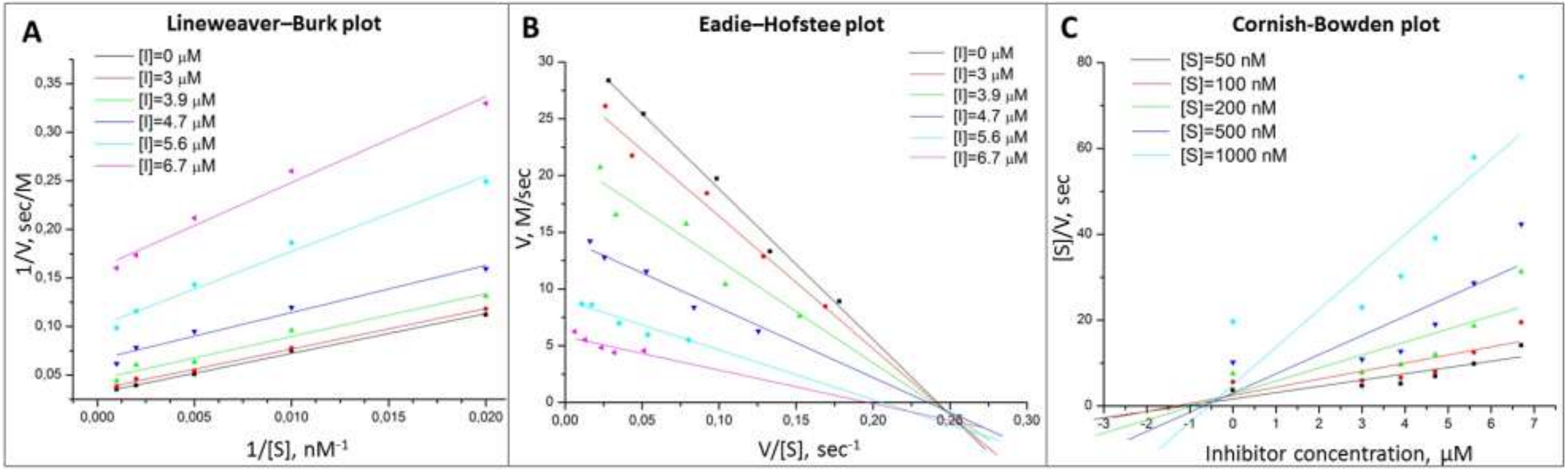

^{13}C-NMR spectrum (400 MHz) of compound 39 in D_2O at 303 K

An important characteristic for 5-iodouracil derivatives is a strong displacement of a C-5 signal in ^{13}C-NMR towards strong magnetic field (72.69 ppm) in comparison with C-5 in uracil and thymine analogues (110-100 ppm).

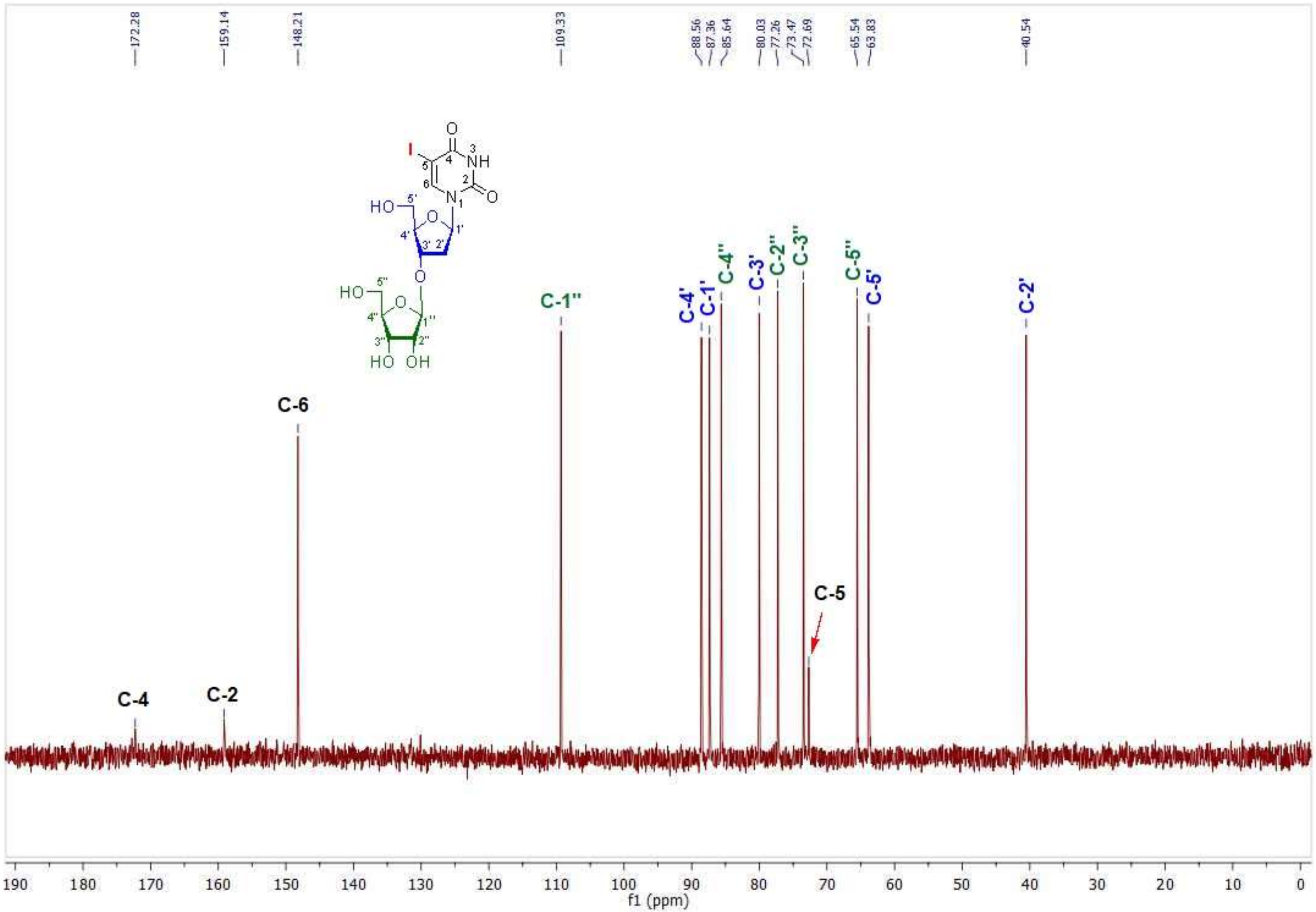

Supplement: Supplemental Material [file IENZ_A_1509210_SM8339.pdf]
